# The impact of different levels of wheat diets on hepatic oxidative stress, immune response, and lipid metabolism in Tibetan sheep (*Ovis aries*)

**DOI:** 10.1186/s12917-023-03874-z

**Published:** 2024-01-18

**Authors:** Boyan Ma, Sayed Haidar Abbas Raza, Sameer D. Pant, Zhanhong Gao, Fengshuo Zhang, Zhiyou Wang, Shengzhen Hou, Mariam Abdulaziz Alkhateeb, Waleed Al Abdulmonem, Yousef Mesfer Alharbi, Abdullah S. M. Aljohani, Linsheng Gui

**Affiliations:** 1 College of Agriculture and Animal Husbandry, Qinghai University, Xining, Qinghai Province 810016 People’s Republic of China; 2 Guangdong Provincial Key Laboratory of Food Quality and Safety/Nation-Local Joint Engineering Research Center for Machining and Safety of Livestock and Poultry Products, South China Agricultural University, Guangzhou, 510642 China; 3 Guangdong Provincial Key Laboratory of Utilization and Conservation of Food and Medicinal Resources in Northern Region, Shaoguan University, Shaoguan, 512005 China; 4 College of Animal Science and Technology, Northwest A&F University, Yangling, Shaanxi 712100 People’s Republic of China; 5 Gulbali Institute, Charles Sturt University, Wagga Wagga, NSW 2678 Australia; 6 Department of Biology, College of Science, Princess Nourah bint Abdulrahman University, P.O. Box 84428, Riyadh, 11671 Saudi Arabia; 7 Department of Pathology, College of Medicine, Qassim University, P.O. Box 6655, 51452 Buraidah, Saudi Arabia; 8 Department of Veterinary Medicine, College of Agriculture and Veterinary Medicine, Qassim University, Buraydah, Saudi Arabia

**Keywords:** Wheat diet, Tibetan lamb, GSEA, RNA-Seq

## Abstract

**Background:**

Compared with corn, wheat contains higher crude protein, amino acids concentration. However, wheat contains a mass of anti-nutritional factors, resulting in increased of the digesta viscosity and impaired the intestinal function in ruminant.

**Objective:**

This study aimed to investigate the effects of substitution of different amounts of wheat for corn on hepatic metabolism in the Tibetan lamb.

**Methods:**

Ninety Tibetan lambs (Body weight = 12.37 ± 0.92 kg) were randomly assigned to three groups: 0% wheat diet (Control), 10% wheat diet (Low group), and 15% wheat diet (High group). The feeding trial lasted for 130 d, including a 10 d adaption period. Hepatic gene expression profiling was performed via RNA sequencing after the conclusion of the feeding trials.

**Results:**

Results showed that greater level of glutathione peroxidase levels in L group compared with those of the C and H groups (*P* < 0.05). The immune indexes, including interleukin-1β (IL-1β), immunoglobulin A (IgA), and IgM were also elevated in L group compared with the other groups (*P* < 0.05). Compared with H group, the hepatocytes were arranged radially, and hepatic plates anastomosed with each other to form a labyrinth-like structure in L group. Transcriptomic analysis showed 872 differentially expressed genes (DEG) between H and L group, of which 755 were down-regulated and 117 were up-regulated. Through Kyoto Encyclopedia of Genes and Genomes (KEGG) enrichment analysis, 32 pathways were significantly enriched (Q-value < 0.05), such as the cAMP signaling pathway, Th1 and Th2 cell differentiation, leukocyte transendothelial migration, platelet activation and adipocytokine signaling pathway. Additionally, the expression of comment DEGs were verified via quantitative reverse-transcription polymerase chain reaction.

**Conclusion:**

In summary, our findings suggest that wheat can be supplemented up to 10% in Tibetan sheep, contributing to improve the hepatic oxidative stress, immune response and lipid metabolism through regulating the expression of related genes.

## Introduction

Tibetan sheep (Ovis aries) is one of the original breeds in China, mainly distributed across Qinghai, Tibet, Gansu and Sichuan provinces, where they inhabit regions with an altitude of over 3000 m. Notably, this species demonstrates remarkable adaptation to the harsh environmental conditions prevalent in these areas, which include extreme cold, low oxygen and strong ultraviolet radiation [1]. Consequently, Tibetan sheep provide a wide variety of resources including meat, milk, fuel, and pelage for the indigenous herdsmen [2].

As the most intricate metabolic organ in mammals, the liver contributes to numerous vital functions, such as digestion, absorption and excretion, material and energy metabolism, immune responses, and detoxification [3]. The liver contributes significantly to gluconeogenesis and the regulation of blood glucose levels [4]. Furthermore, liver sinusoidal cells are responsible for removing harmful metabolites and exogenous microorganisms, thereby maintaining immune balance [5]. Therefore, the nutrient metabolism of the liver is crucial to the growth and development of livestock.

As the breeding industry has rapidly evolved, a significant challenge has emerged: the scarcity of corn resources, now a critical issue in animal husbandry. In the year 2022, there was a notable 7.0% rise in the average price of corn. In contrast, wheat’s average price saw a decrease of 3.76%. This disparity led to a major shift in China, where the breeding industry turned to wheat, using nearly 45 million tons—a staggering 95.7% increase from previous years. Meanwhile, in Europe, wheat has long been the go-to energy feed for animal husbandry, further cementing its status as the preferred alternative to corn.

In comparison to maize, wheat contains approximately 5% more crude protein and is second only to that of maize in terms of its effective energy content. This makes it a promising alternative to maize feed [6]. While both wheat and maize have significant starch content, the rumen only digests around 60% of maize starch, compared to over 90% of wheat starch [7]. Previous research has shown that increasing the dietary wheat ratio in livestock did not result in significant differences in growth performance and apparent nutrient digestibility [8]. However, it is important to note that wheat contains a high concentration of anti-nutritional factors, especially araboxylan. These factors increase the digesta viscosity in the gut, potentially impairing ruminant intestinal function [9]. Therefore, identifying an appropriate wheat replacement ratio is crucial for optimal livestock development and growth.

However, little attention has been paid to the effect of dietary wheat on liver of ruminant. In light of the above considerations, the objective of this study was to evaluate the impact of varying wheat proportions in the diet, on hepatic oxidative damage, immune response, and lipid metabolism in the Tibetan sheep by RNA-Seq sequencing technology.

## Materials and methods

### Ethical statement

All animal procedures for experiments were approved by the Committee of Experimental Animal care and handling techniques were approved (QUA-2020–0710) by the Qinghai University of Animal Care Committee. Moreover, all applicable rules and regulation of the organization and government were followed regarding the ethical use of experimental animals.

### Animal diet and sample collection

A total of 90 healthy male Tibetan lambs with similar body weight (12.37 ± 0.92 kg) and physiological state were selected. According to dietary treatments, the lambs were randomly divided into three treatment groups, including a control group (100% corn), 10% wheat group (10% substitution for corn), 15% wheat group (15% substitution for corn), and 100% wheat group (100% substitution for corn) on a DM basis. This feeding trial was conducted at a commercial sheep farm in Gonghe County, Qinghai Province, China (Coordinate 100^o^75’N, 35^o^57’E, altitude at 3,370 m). All three diets contained approximately 30% forage and 70% concentrate (Table 1) and included 1:1 ratios of silage grass and oat hay. Daily nutrient requirements were met for all lambs (NRC, 2007) [10]. Fresh drinking water and feed were provided *ad* libitum. The adaptation period for the diet changes lasted 10 d, and the study continued for 120 d. At the end of the experiment, nine sheep, with three replicates each, were randomly selected and rendered unconscious with a stunning machine before being bled to death by neck bleeding (Stun machine model: AOSH 2000, AOSH Automation Technology Company, Wuxi, China). After slaughter, liver samples were taken and placed immediately into liquid nitrogen for RNA extraction, while the remaining tissue samples were fixed in 4% paraformaldehyde for tissue sectioning.

**Table 1:** Composition and nutrient levels of basal diets (DM basis) %

Items	C group	L group	H group
Oaten hay	15.00	15.00	15.00
Oats silage	15.00	15.00	15.00
Corn	45.85	40.04	37.10
Soybean meal	5.60	3.50	5.25
Rapeseed meal	11.20	10.85	10.15
Cottonseed meal	2.80	4.06	2.45
Wheat	0.00	7.00	10.50
NaCl	0.35	0.35	0.35
Limestone	0.70	0.70	0.70
Premix^a^	3.50	3.50	3.50
Total	100.00	100.00	100.00
Nutrient levels			
DE/(MJ/kg)	11.78	11.77	11.81
Dry matter	85.82	85.82	85.80
Crude protein	15.26	15.06	15.11
Crude fat	2.90	2.78	2.75
Neutral detergent fiber	25.54	25.87	25.79
Acid detergent fiber	16.73	16.74	16.58
Starch	32.59	32.24	32.80
Ash	4.69	4.66	4.66

*Note*: ^a^Provided per kilogram of diets: Cu 15 mg, Fe 55 mg, Zn 25 mg, Mn40 mg, Se 0.30 mg, I 0.5 mg, Co 0.20 mg, VA 20 000 IU, VD 4 000 IU, VE 40 IU

### Chemical analysis

The dry matter (DM) content was determined by drying the feeds at 105 °C in a forced-air oven for 4 h. The CP (ID number 968.06), ether extract (EE) (ID number 920.39), and ash (ID number 942.05) contents in diet were measured as proposed by the Association of Official Analytical Chemists (1990). Neutral detergent fiber (NDF) was analyzed as proposed by Licitra et al. (1996) [11]. Acid detergent fiber (ADF) was determined using the methods described by Van Soest et al. (1991) [12].

### Antioxidant and immune response index assays

Superoxide dismutase (SOD), total antioxidant capacity (T-AOC), glutathione peroxidase (GSH-Px), and malondialdehyde (MDA) values of hepatic oxidative stress parameters in sheep were analyzed by using Elisa Kit. IL-1β, TNF-α, immunoglobulin A (IgA), IgG, and IgM values of hepatic immune indices were also analyzed by using Elisa Kit. All ELISA kits used in this study were procured from Jiancheng Bioengineering Institute (Nanjing, China).

### Histological analysis of the liver

Tissues were fixed, paraffin-embedded, and sectioned (5 μm) with a rotary microtome, as per the protocol outlined by Prophet et al. (Prophet et al., 1992). Hematoxylin and eosin were used to stain the tissue sections. The stained slides were evaluated using a DP2-BSW digital microscope (Olympus, Tokyo, Japan) at 40 × magnification. Cross-sectional areas of the liver were determined using Image-Pro Plus 6.0 software (Media Cybernetics Inc., Bethesda, MD, USA).

### RNA-Seq analysis

Total RNA was extracted using TRIzol (Thermo Fisher Scientific, Waltham, MA, USA) and was quantified by the 260/280 nm absorbance ratio using a NanoDrop ND2000 spectrophotometer (Thermo Fisher Scientific, Waltham, MA, USA). The integrity of RNA and the library was examined using an Agilent 2100 Bioanalyzer (Agilent Technologies, Santa Clara, CA, USA). The final cDNA libraries were constructed using Illumina NovaseqTM 6000 following the vendor’s recommended protocol. The raw reads with low-quality reads, adaptors, and highly unknown base N content were trimmed using SOAPnuke, a filtering software developed by BGI. Then, clean reads were aligned to the sheep reference genome of *Ovis aries* (Oar_Version 3.1) using HISAT2 (Version 2.1.0).

DESeq2 package in R were used to normalize read counts and detect differentially expressed genes (DEGs) at a False Discovery Rate (FDR) threshold of 0.05. Genes with |log2Fold Change|> 0.5 and Q-values < 0.05 were recognized as DEGs.

The Kyoto Encyclopaedia of Genes and Genomes (KEGG) pathways were performed using the phyper package in R (Version 3.6.2). After FDR correction, a Q-value < 0.05 was used as a threshold. All analyses with a Q-value < 0.05 were considered significantly enriched. The gene set enrichment analysis (GSEA) was performed by GSEA software (Version 4.1.0) on three sets of genes (IL-6-JAK-STAT3-PATHWAY, REACTIVE-OXYGEN-SOECIES-PATHWAY, FATTY-ACID-METABOLISH) screened from the GSEA database. Additionally, the protein–protein interaction (PPI) network was created by Cytoscape software (Version 3.8.0).

### Quantitative reverse transcription polymerase chain reaction (qRT-PCR)

Nine DEGs were randomly selected for qRT-PCR to verify the accuracy of the transcriptome sequencing data. The mRNA expression of the DEGs was normalized to the expression of the housekeeping gene glyceraldehyde 3-phosphate dehydrogenase (GAPDH) in the different samples. The primers used for this study were designed using an online primer design tool and the sequences are presented in Table 2. Relative expression of genes was calculated by the 2^−ΔΔCt^ method.

**Table 2:** Primers used in qRT-PCR

Gene	GenBank accession	Primer sequence (5’-3’)	Tm (°C)	Product length
SDHC	XM_004002697.4	F-TCCGACACTTGATGTGGGACCTAG R-ACAACACAGTAAGAACCAGGACAGC	60.0	90 bp
NDUFS1	XM_004004855.4	F-TTTGGGAACTACAGGCAGAGGAAAC R-AAGGCATAGGGCTTAGAGGTCAGG	60.0	107 bp
MDH2	XM_004021260.5	F-GCGATGAACGGAAAGGAAGGAGTC R-AGCAGCAACGGTGTGGAGAAATAC	60.0	89 bp
CXCL10	NM_001009191.1	F-GCATACCTCTCTCTAGGAACACACG R-GGGCAGGATTGACTTGCAGGAATC	60.0	112 bp
TNF	XM_012100437.4	F-CAACGGCGTGGAGCTGAAAGAC R-TGAAGAGGACCTGCGAGTAGATGAG	60.0	80 bp
TYK2	XM_042249928.1	F-TGGTGAGATGGTAGCCGTGAAGG R-ACTTGACGATGTGCTTGTGGTAGAG	60.0	116 bp
ACADL	XM_027965150.2	F-TTGAAGATGTACGGTTGCCAGCAG R-TGGAAGCTCTTGCATGAGGTAATGG	60.0	80 bp
HSD17B4	XM_004008685.4	F-GAGGTTGGAGCAGGATGGATTGG R-CTCTGAGGCTTGGTGGCATTATCG	60.0	149 bp
HIBCH	XM_004004498.5	F-AAAGGCTTTCTGTGCTGGAGGTG R-CTGGCAAGAATCAATGGCGTTGTTC	60.0	124 bp
GAPDH	NM_001190390.1	F- CGGCACAGTCAAGGCAGAGAAC R- CACGTACTCAGCACCAGCATCAC	60.0	115 bp

### Correlation analysis

Correlation network analysis was performed using the OmicStudio tools. The data for correlation analysis were derived from ELISA and fluorescence quantitative measurement results.

## Results

### Antioxidant and immune response index assays

As shown in Table 3, the indicators of glutathione peroxidase in L group demonstrated a statistically significant increase compared to the C and H groups (*P* < 0.05). Furthermore, the indicators of interleukin-1β, immunoglobulin (Ig)A, and IgM in L group were significantly elevated compared with the levels in the other two groups (*P* < 0.05).

**Table 3:** Antioxidant and immune indexes assay

Items	C group	L group	H group	*P*-value
GSHPx	0.81 ± 0.04^b^	1.01 ± 0.06^a^	0.83 ± 0.05^b^	0.02
MDA	1.02 ± 0.03	1.08 ± 0.01	1.05 ± 0.03	0.23
SOD	1.11 ± 0.10	1.74 ± 0.37	1.06 ± 0.07	0.09
T-AOC	1.18 ± 0.05	1.30 ± 0.05	1.16 ± 0.02	0.06
TNFα	1.39 ± 0.01	1.41 ± 0.02	1.36 ± 0.01	0.08
IL-1β	1.51 ± 0.01	1.51 ± 0.01	1.48 ± 0.01	0.11
IgA	1.48 ± 0.01^b^	1.51 ± 0.01^a^	1.45 ± 0.01^b^	0.01
IgM	1.12 ± 0.02^b^	1.19 ± 0.01^a^	1.11 ± 0.02^b^	0.00
IgG	0.59 ± 0.04	0.65 ± 0.04	0.59 ± 0.04	0.39

^a,b^Means in the same row with different superscripts differed (*P* < 0.05)

### Histological analysis of the liver

**Fig. 1:**
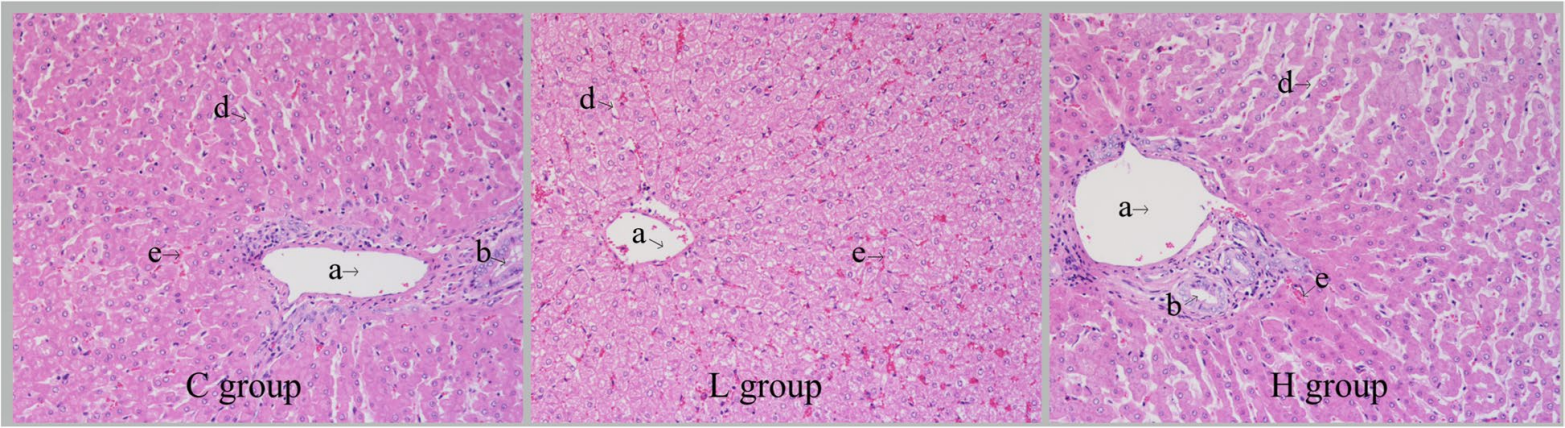
Liver tissue frozen sections. HE staining, 400 x. **a** Interlobular veins. **b** Interlobular artery. **d** Sinus periphery gap. **e** Hepatic blood sinusoids

As illustrated in Fig. 1, the hepatocytes in the L group displayed a radial arrangement, forming hepatic plates that were centered around the central vein. Additionally, these plates anastomosed with each other, forming a lost-like structure. On the other hand, in the H group, hepatic lobules were indistinguishable from the surrounding liver tissue, and the hepatic plates were separated from each other, resulting in a loosely distributed arrangement of hepatocytes.

### Characterization of RNA-Seq data

**Fig. 2:**
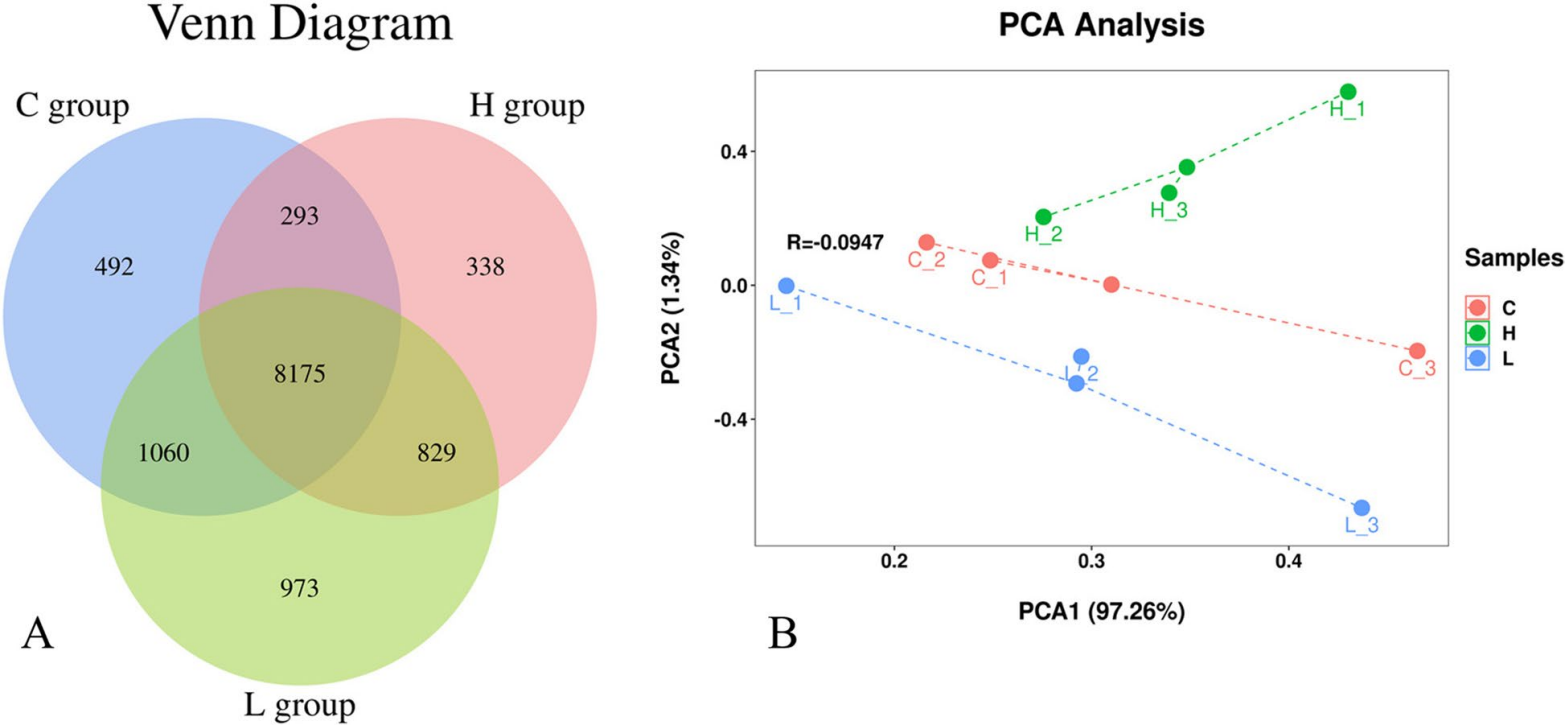
**A** Gene Expression Venn Diagram. **B** PCA Analysis Diagram

Mapping of the reads obtained from sequencing against the *Ovis* reference genome resulted in between 40 and 51 million reads per sample, with an average of 43 million reads. Quality control measures were applied to ensure that the Q30 (%) score was ˃98% for each sample, while the GC content (%) was > 45%. The mapping rate for reads were ˃90%, with an average of 92.36%. The nonsplice reads were > 37.57%, and the percentage of multi-mapped reads was 60% (average = 59.83%). Additionally, a total of 12160 genes were obtained based on the similarity > 97%. Of those, 492 genes were unique to the C group, 973 genes were unique to the L group, 338 genes were unique to the H group (Fig. 2A). Based on Binary-Jaccard algorithm, the first two axes of the PCA1 explained 97.26% and 1.34% of the variation and the two principal components covered 98.60% of the variation (Fig. 2B).

### DEGs identification and KEGG pathways analyses

**Fig. 3:**
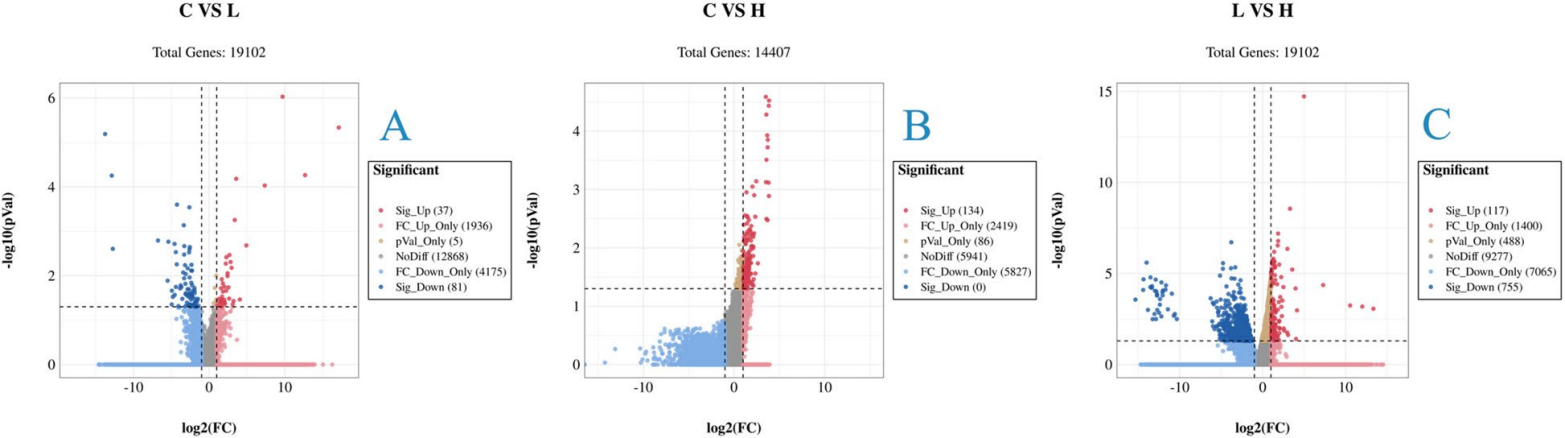
Volcano plot displaying DEGs between the C and L groups (**A**), between the C and H groups (**B**), and between the L and H groups (**C**). Sig-Up: Significantly upregulated genes. FC-Up-Only: Only FC values differ to upregulate genes. pval-Only: Only P-value difference genes. NoDiff: no significantly different genes. FC-Down-Only: Only FC values differ in down-regulated genes. Sig-Down: Significantly downregulated genes

A total of 1346 DEGs were identified according to the Q values (< 0.05). Specifically, in C vs. L, 37 genes were upregulated and 81 genes were downregulated (Fig. 3A). In C vs. H, 134 genes were upregulated (Fig. 3B). In L vs. H, 117 genes were upregulated and 755 genes were downregulated (Fig. 3C). KEGG pathway analysis revealed that the common DEGs were enriched in 196 pathways and significantly enriched in 32 pathways. A number of significantly enriched pathways, including cAMP signaling pathway, Th1 and Th2 cell differentiation, leukocyte transendothelial migration, platelet activation and adipocytokine signaling pathway were related to immune response and lipid metabolism (*P* < 0.05).

### GSEA and PPI network analyses

**Fig. 4:**
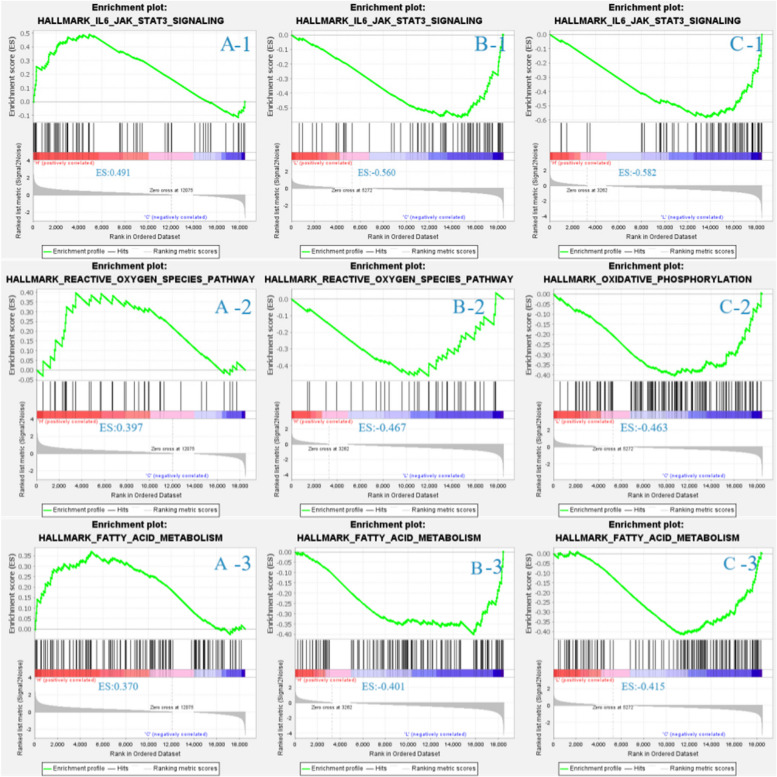
Gene set enrichment analysis (GSEA) between the C and L groups (**A**), between the C and H groups (**B**), and between the L and H groups (**C**). 1 represent for the IL6-JAK-STAT3-SIGNALING gene set. 2 represent for is REACTIVE-OXYGEN-SPECIES-PATHWAY gene set. 3 represent for FATTY-ACID-METABOLISM gene set

Three background gene sets, including IL-6-JAK-STAT3-PATHWAY, REACTIVE-OXYGEN-SOECIES-PATHWAY and FATTY-ACID-METABOLISH, were selected and analyzed (Fig. 4). The results revealed that the L group exhibited positive enrichment in all three gene sets when compared with the C group, while the H group exhibited negative enrichment in the gene sets.

**Fig. 5:**
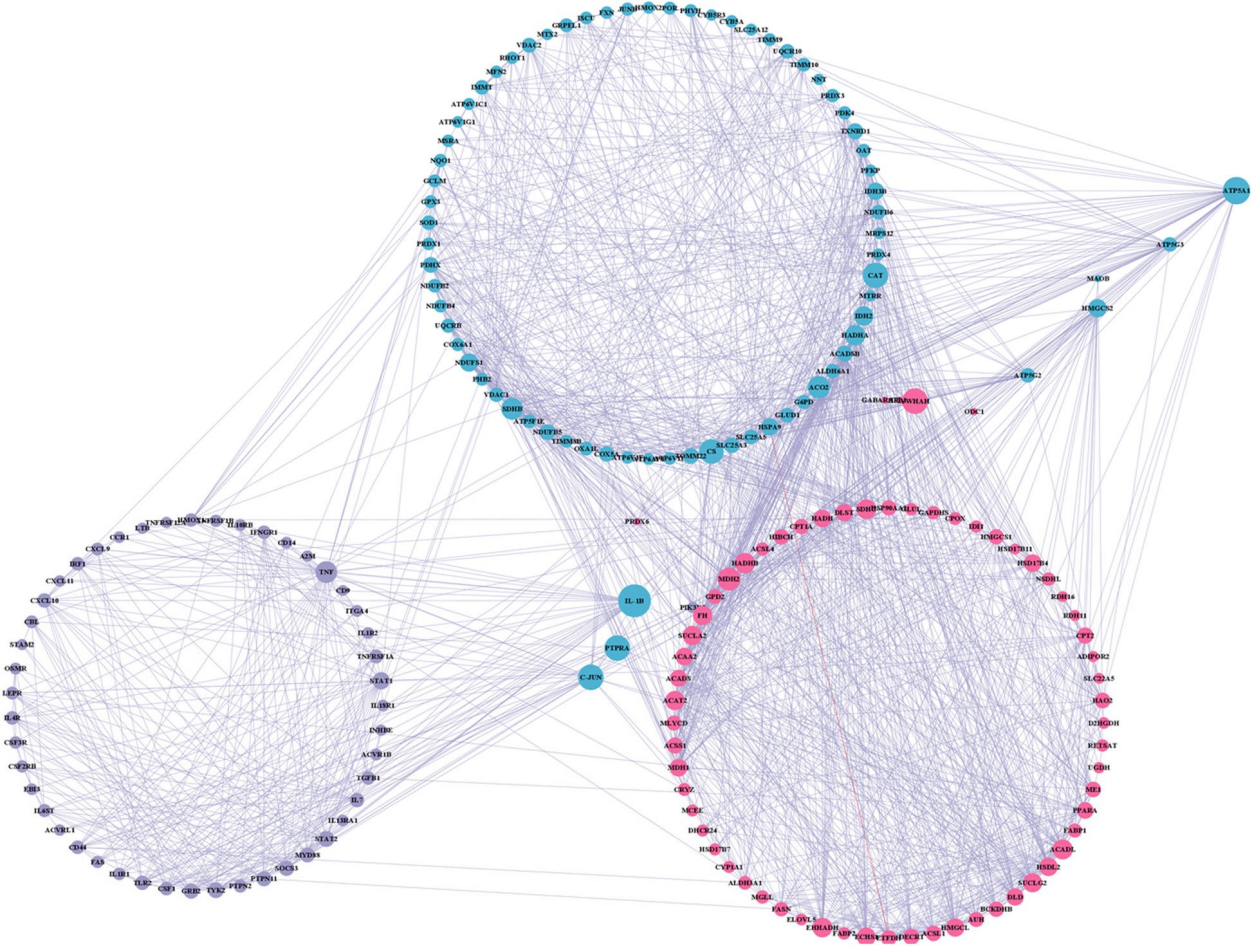
Protein–protein interaction (PPI) network. Red represents the FATTY-ACID-METABOLISM gene set, blue represents the REACTIVE-OXYGEN-SPECIES-pathway gene set, and purple represents the IL6-JAK-STAT3-SIGNALING gene set. The network diagram’s circle size indicates the number of nodes that are linked, and the greater the circle's diameter, the more nodes that are connected

PPI network interaction analysis was conducted for further evaluation of the differential gene relationships and identification of the hub genes. The results, as illustrated in Fig. 5, revealed that the top 10 hub genes were interleukin 1 beta (*IL-1B*), protein tyrosine phosphatase receptor type A (*PTPRA*), transcription factor AP-1-like (*C-JUN*), tyrosine 3-monooxygenase/tryptophan 5-monooxygenase activation protein eta (*YWHAH*), phosphoinositide-3-kinase regulatory subunit 5 (*PIK3R5*), ATP synthase 5a1 (*ATP5A1*), mitochondrial Fo complex subunit C3 (*ATP5G3*), 3-hydroxy-3-methylglutaryl-CoA synthase (*HMGCS*), mitochondrial Fo complex subunit C3 (*ATP5G3*), and ornithine decarboxylase 1 (*ODC1*).

### Correlation of phenotypic data with sequencing data and verification of qRT-PCR

**Fig. 6:**
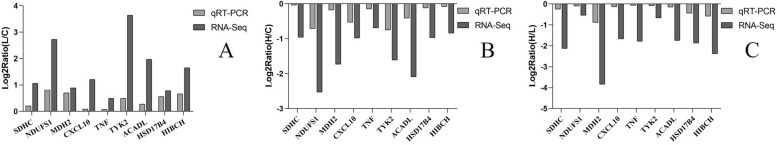
Verification of the differentially expressed genes by real-time quantitative polymerase chain reaction (qRT-PCR). **A** Compare the genes in the C and the L groups. **B** Compare the genes in the C and the H groups. **C** Compare the genes in the L and the H groups

Nine functional DEGs, including succinate dehydrogenase complex subunit C (*SDHC*), NADH:ubiquinone oxidoreductase core subunit S1 (*NDUFS1*), malate dehydrogenase 2 (*MDH2*), C-X-C motif chemokine ligand 10 (*CXCL10*), tumor necrosis factor (*TNF*), tyrosine kinase 2 (*TYK2*), acyl-CoA dehydrogenase long chain (*ACADL*), hydroxysteroid 17-beta dehydrogenase 4 (*HSD17B4*) and 3-hydroxyisobutyryl-CoA hydrolase (*HIBCH*), were selected for qRT-PCR verification of the RNA-Seq data. The results revealed consistent correlations between the RNA-Seq results and the mRNA levels measured by qRT-PCR (Fig. 6).

**Fig. 7:**
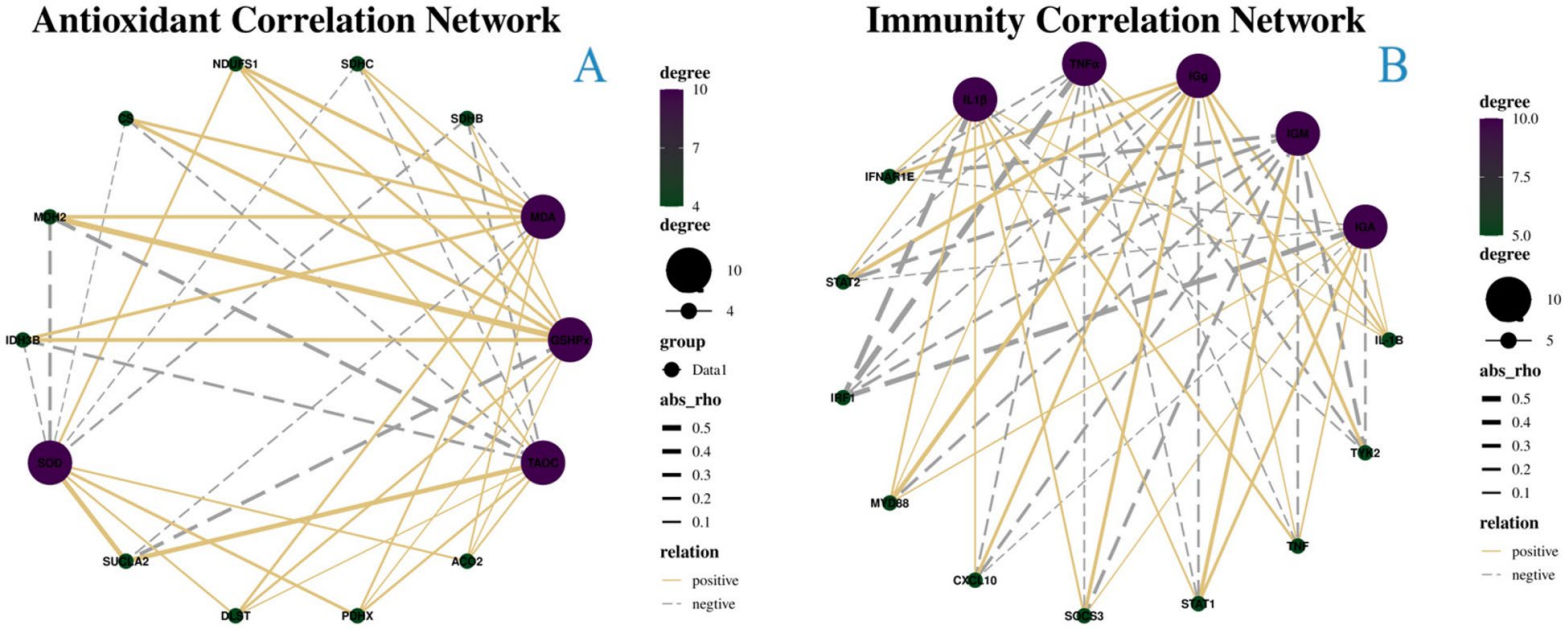
Correlation Network of antioxidant correlation (**A**) and immunity correlation (**B**). The number of nodes connected to the gene increases with increasing purple color, whereas nodes decrease with increasing green color. abs-rho: demonstrates the degree of association. The silver dotted line denotes a negative connection, whereas the solid gold line denotes a positive correlation

In addition, the association between antioxidant and immune response index data, and the level of gene expression identified through sequencing was also investigated. The results of this analysis revealed that MDA, GSH-Px, T-AOC, SOD, IL-1, TNF, IgG, IgM, and IgA were substantially connected with the stability and sequencing data as well as with the accuracy of the number of sequencing data (Fig. 7).

## Discussion

The liver is an important organ and has very pivotal physiological functions, such as nutrient metabolism, detoxification and absorption [13]. However, excessive intake of nutrients or dietary components led to damage to the liver. It was found that a high-fat diet resulted in severe steatosis, swelling of hepatocytes, and a significant increase in apoptosis in rats [14]. Similarly, high starch in diet caused leds to disruption of hepatic glycogen metabolism and liver fibrosis via mediating PI3K/Akt signaling pathway [15]. Additionally, high rumen degradable starch diet significantly reduced rumen pH and acetate-to-propionate ratio in dairy goats, and increased free lipopolysaccharides (LPS) in rumen fluid and plasma and induced inflammatory response in liver [16]. Our results showed that the histoarchitecture of hepatic cells depicted greater cell damage when fed 15% dietary wheat. One possible explanation is that, high inclusion of wheat in diet resulted in increases of LPS concentration in digestive tract [17], which translocated into the liver and induced hepatic damage.

Redox homoeostasis plays an important role in maintaining normal physiological functions in mammals. Oxidative stress results from an unbalance of oxidoreduction [18]. In the present study, no significant difference of MDA, SOD and T-AOC were observed in liver among those treatments, while GSHPx concentration was significantly higher in sheep fed 10% wheat diets compared with the control sheep. It was generally supposed that higher activity of GSHPx enhanced the free-radical scavenging capacity [19]. On the other hand, the immune system is closely involved in the maintenance of cellular homeostasis. Among the five Igs, IgA was involved in antisepsis, sterilization, and antivirus [20]. IgM is the first indicator of the immune response, contributing to bacterial lysis and hemolysis [21]. The remarkable affects in IgM and IgA were observed when fed 10% wheat diets in the current study. This suggests that 10% wheat supplementation in the diet tended to increase oxidation resistance and immunity in liver, thereby promoting health status for Tibetan sheep.

Recent studies have attempted to identify the genes associated with liver and understand the underlying mechanisms of ovine liver metabolism function. By combining the RNA-seq results of Hu sheep and Tibetan sheep, Li et al. (2022) identified 1179 DEGs in liver, including 25 fat-metabolism-related genes [22]. Peng, et al. (2022) found that 2188 DEGs were markedly up or downregulated between muscle and liver. There were 950,160 upregulated and 100,771 downregulated genes in the liver and muscle, respectively [23]. In the current study, 1346 genes were found to be differentially expressed in the subcutaneous fat of sheep fed different levels of wheat diets of Tibetan sheep.

Nine of these DEGs, including *SDHC*, *NDUFS1*, *MDH2*, *CXCL10*, *TNF*, *TYK2*, *ACADL*, *HSD17B4* and *HIBCH* were identified as candidate genes that might be involved in regulating hepatic oxidative stress, and immune response. For the oxidative stress, *SDHC* mutation was significantly increased oxidative stress, resulting in cellular apoptosis and tumorigenesis [24]. *NDUFS1* regulated the oxidative stress by altering the mitochondrial reactive oxygen species formation [25]. *SIRT3* knockdown changed the acetylation status of *NDUFS1*, which induced altered mitochondrial oxidative phosphorylation, and eventually defects in the cellular insulin signaling pathway [26]. *MDH2* produced oxaloacetate was a metabolic switch rewiring the fuelling of respiratory chain and tri-carboxylic acid cycle [27]. For the immune response, miR-15a expression facilitates proinflammatory cytokines production and contributes to immune response at least in part via regulating *CXCL10* expression [28]. *TNF* was an important pro-inflammatory cytokine in the innate immune systems, which was primarily produced by macrophages in response to microbial exposures including lipopolysaccharide [29]. *TYK2* belonged to the JAK family and acts as an intermediary between cytokine receptors and STAT transcription factors, which involving in immune and inflammatory signaling [30]. As a key enzyme that regulates β-oxidation of long-chain fatty acyl-CoAs, *ACADL* prevented immune evasion by targeting Hippo/YAP signaling [31]. Currently, our analysis revealed consistent correlations between the RNA-Seq results and the mRNA levels measured by qRT-PCR. We speculated that 10% dietary wheat significantly decreases the hepatic oxidative stress and immune response of Tibetan sheep by modulating the expression of related functional genes.

## Conclusions

The present study was designed to investigate the impact of three levels of wheat substitution for corn on hepatic metabolism. The results showed that as the amount of wheat included in the diet increased, there were irregular changes in the arrangement of hepatocytes, with the 10% wheat-added group having more tightly-packed hepatocytes. Furthermore, significant upregulation of genes related to oxidative stress, immune response, and fatty acid metabolism was observed in the 10% wheat-supplemented group. These results provide significant insight into the effects of various wheat supplementation levels on hepatic metabolism in Tibetan lamb.

## Data Availability

The datasets presented in this study can be found in online repositories. The names of the repository/repositories and accession number(s) can be found below: NCBI SRA (accession: SAMN35795877) (https://www.ncbi.nlm.nih.gov/sra/).
